# iModulonDB 2.0: dynamic tools to facilitate knowledge-mining and user-enabled analyses of curated transcriptomic datasets

**DOI:** 10.1093/nar/gkae1009

**Published:** 2024-11-04

**Authors:** Edward A Catoiu, Jayanth Krishnan, Gaoyuan Li, Xuwen A Lou, Kevin Rychel, Yuan Yuan, Heera Bajpe, Arjun Patel, Donghui Choe, Jongoh Shin, Joshua Burrows, Patrick V Phaneuf, Daniel C Zielinski, Bernhard O Palsson

**Affiliations:** Department of Bioengineering, University of California, San Diego, La Jolla, CA 92101, USA; Department of Bioengineering, University of California, San Diego, La Jolla, CA 92101, USA; Department of Bioengineering, University of California, San Diego, La Jolla, CA 92101, USA; Department of Bioengineering, University of California, San Diego, La Jolla, CA 92101, USA; Department of Bioengineering, University of California, San Diego, La Jolla, CA 92101, USA; Department of Bioengineering, University of California, San Diego, La Jolla, CA 92101, USA; Department of Bioengineering, University of California, San Diego, La Jolla, CA 92101, USA; Department of Bioengineering, University of California, San Diego, La Jolla, CA 92101, USA; Department of Bioengineering, University of California, San Diego, La Jolla, CA 92101, USA; Department of Bioengineering, University of California, San Diego, La Jolla, CA 92101, USA; Department of Bioengineering, University of California, San Diego, La Jolla, CA 92101, USA; The Novo Nordisk Foundation (NNF) Center for Biosustainability, The Technical University of Denmark, Kongens Lyngby 2800, Denmark; Department of Bioengineering, University of California, San Diego, La Jolla, CA 92101, USA; Department of Bioengineering, University of California, San Diego, La Jolla, CA 92101, USA; The Novo Nordisk Foundation (NNF) Center for Biosustainability, The Technical University of Denmark, Kongens Lyngby 2800, Denmark

## Abstract

iModulons—sets of co-expressed genes identified through independent component analysis (ICA) of high-quality transcriptomic datasets—provide an unbiased, modular view of an organism's transcriptional regulatory network. Established in 2020, iModulonDB (iModulonDB.org) serves as a centralized repository of curated iModulon sets, enabling users to explore iModulons and download the associated transcriptomic data. This update reflects a significant expansion of the database—19 new ICA decompositions (+633%) spanning 8 925 expression profiles (+1370%), 503 studies (+2290%) and 12 additional organisms (+400%)—and introduces new features to help scientists decipher the mechanisms governing prokaryotic transcriptional regulation. To facilitate comprehension of the underlying expression profiles, the updated user-interface displays essential information about each data-generating study (e.g. the experimental conditions and publication abstract). Dashboards now include condition-specific coloring and highlight data generated from genetically perturbed strains, enabling users to rapidly interpret disruptions in transcriptional regulation. New interactive graphs rapidly convey omics-derived indicators (e.g. the explained variance of ICA decompositions, genetic overlap between iModulons and regulons). Direct links to operon diagrams (BioCyc) and protein-protein interaction networks (STRING) provide users with seamless access to external resources for further assessment of iModulons. Lastly, a new suite of search-driven and species-wide analysis tools promotes user-engagement with iModulons, reinforcing iModulonDB’s role as a dynamic, interactive knowledgebase of prokaryotic transcriptional regulation.

## Graphical Abstract

**Graphical Abstract:**
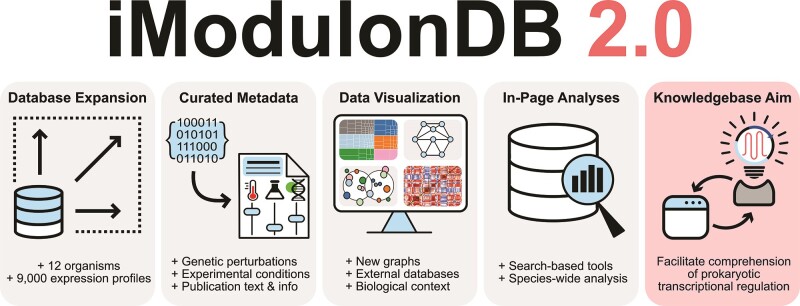

## Introduction

*iModulons*—first introduced by Sastry *et al.* (1)—are sets of independently-modulated (co-expressed) genes identified through independent component analysis (ICA) of high-quality transcriptomic data. ICA decomposes a transcriptomic dataset (i.e. a matrix of gene expression profiles across multiple experiments) into an *iModulon matrix* (representing the weights of all genes in each iModulon) and an *activity matrix* (indicating the activity of each iModulon across all conditions) (see Supplementary Figure S1). To validate this omics-driven representation of an organism's transcriptional regulatory network (TRN), iModulons are often compared to their corresponding *regulons*—sets of co-regulated genes confirmed by experimental measurement (i.e. shared transcription factor binding). In addition to capturing well-characterized regulons, iModulons often provide deeper insights into an organism's TRN.

First, iModulons offer an unbiased global view of an organism's TRN, especially when experimental validation is limited (e.g. in *A. baumannii* (2), *L. reuteri* (3), *P. syringae* (4), *S. acidocaldarius* (5), *S. pyogenes* (6) and *S. elongatus* (7)). In well-characterized organisms, iModulons can identify genes outside their associated regulons that are prime candidates for experimental validation (e.g. expansion of the MetJ and CysB regulons in *E. coli* (1)). Additionally, when a transcription factor (TF) is co-modulated with genes it is not known to regulate, iModulons can reveal potential regulatory relationships (e.g. BrlR-mediated regulation of the *mexPQ-ompE* multi-drug efflux system in *P. aeruginosa* (8)). Second, iModulons help assign putative functions to uncharacterized genes that are co-modulated with known genes (e.g. 224 genes in *E. coli* (1)), and reveal new roles for genes under novel conditions (e.g. activation of tryptophan synthesis during ethanol stress in *B. subtilis* (9)). Third, activated iModulons in TF knock-out experiments reveal the gene targets of the deleted TF (e.g. uncharacterized TFs in *E. coli* (1)). Fourth, iModulons modularize TRNs, simplifying our understanding of complex regulatory processes (e.g. three iModulons coordinate the regulation of 65 virulence factors in *S. aureus* (10)). Lastly, when individual expression profiles are contextualized within a larger compendium suitable for ICA, iModulons can offer insights into global cellular responses across different environments (e.g. host-cell specific activation of multiple iModulons during *M. tuberculosis* infection (11)).

As independent component analysis emerged as powerful tool for analysing an increasing number of available prokaryotic transcriptomes, iModulonDB (https://iModulonDB.org) was released in 2020 as a knowledgebase of curated sets of iModulons, enabling users to interact with iModulons and download the underlying transcriptomic datasets (12). Curated iModulon sets published on iModulonDB are generated using a consistent, publicly available pipeline (iModulonMiner (13)) for gathering and processing RNA-seq data into a larger transcriptomic compendium, computing iModulons, and formatting dashboard files (see Supplementary Figure S2 for the website architecture). As of this publication, datasets published on iModulonDB have been cited over 300 times and the website maintains a user base of approximately 500 monthly active users.

**Figure 1.**
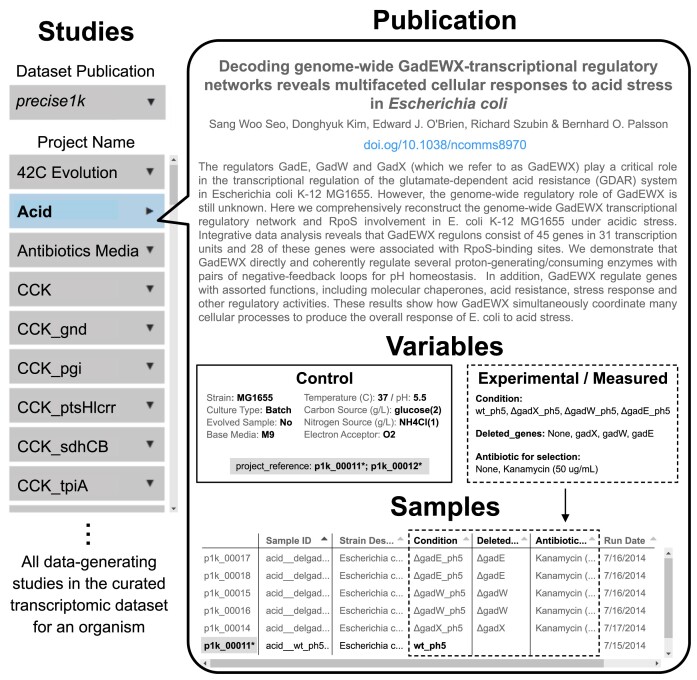
The ‘Projects’ page displays pertinent data associated with each study. When available, the title, authors, DOI, and abstract of the associated publication are displayed. Control and experimental variables and the reference condition are highlighted. The samples table reflects the experimental conditions of each expression profile. The ‘Acid’ study (24) in the *E. coli* PRECISE-1K dataset (17) is shown.

**Figure 2.**
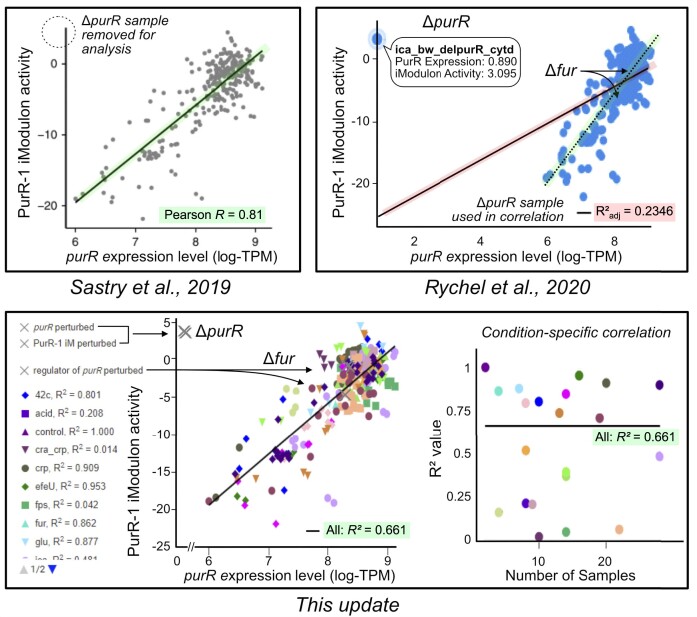
Curation of genetic perturbations and sample conditions improves interpretability of iModulons. The *purR* gene is a regulator of the PurR-1 iModulon in the *E. coli* PRECISE-278 dataset. The PurR-1 iModulon activity and purR gene expression is plotted from the original ICA publication^1^ (Sastry *et al.*, 2019), the iModulonDB dashboards in the initial release^12^ (Rychel *et al.*, 2020) and from this update. (top left) Analysis of this iModulon-regulator pair examines if the iModulon activity is correlated with its regulator's gene expression. Samples from purR mutant strains are removed from the analysis (green) of the PurR-1 iModulon. (top right) Raw data is displayed and used to calculate correlations in the initial release of iModulonDB. Here, ΔpurR samples disrupt the analysis (red). (bottom left) Samples containing genetic perturbations are highlighted (‘X’) and excluded from the analysis. (bottom right) Correlations are also presented in a condition-specific manner. Note: graphs have been slightly modified to emphasize key differences.

iModulonDB’s initial release included iModulon dashboards and transcriptomic datasets for three organisms: *E. coli* (1), *B. subtilis* (9), *S. aureus* (10). In this update, we have added *19 new transcriptomic datasets spanning 12 additional organisms* (Table 1). This update also introduces various features developed to facilitate users’ understanding of the information-rich content of iModulonDB. The abstracts—as well as the control and experimental variables—of each study used to generate RNA-seq data are now displayed on a new ‘Projects’ page (Figure 1). Dashboards in the initial release displayed raw data, requiring careful interpretation of the resulting iModulons. To alleviate this burden and provide users with an immediate and accurate assessment of the conditions that affect iModulon activity and/or gene expression, graphs are now color-coded by project, genetically perturbed samples are highlighted, correlations are calculated without genetically perturbed samples, and condition-specific correlations are plotted (Figure 2). Biological context for iModulons is provided with direct links to the operon diagrams (via BioCyc (14)) and full protein-protein interaction network graphs (via STRING (15)) (Supplementary Figure S5). New graphs enhance the global characterization of iModulon datasets; TreeMaps display the explained variance of the ICA decomposition, while Regulon Recall plots compare the gene overlap between iModulons and reported regulons (Supplementary Figure S3). The overlap between an iModulon and its corresponding regulon is further detailed by the gene expression correlation matrix heatmap on each ‘iModulon’ page (Supplementary Figure S4).

**Table 1.** Database size of iModulonDB

	Initial Publication^1^ (2020)	This Update (2024)
**Datasets**	3	22
**Organisms**	3	15
**Samples**	651	9576
**iModulons**	204	1924
**Studies**	22	525

Datasets: number of ICA decompositions. Some organisms are associated with multiple datasets. Samples: number of expression profiles. iModulons: number of iModulons, each with their own page on the site. Studies: number of unique experiments associated with the original generation of samples, accompanying publication is displayed on the ‘Projects’ page when available.

**Figure 3.**
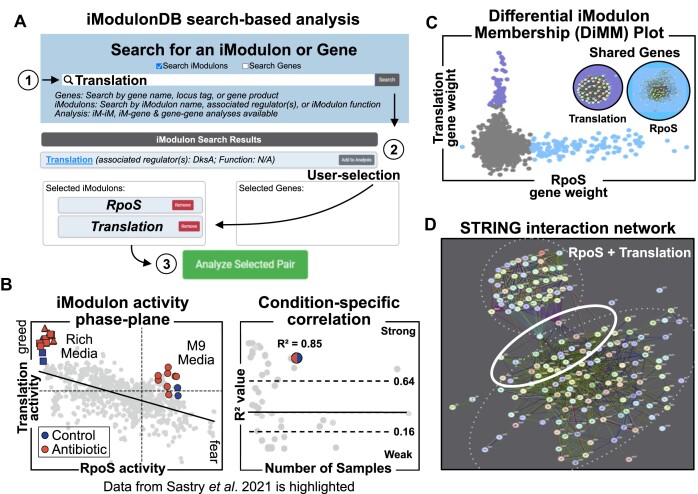
Search-driven analysis of the RpoS and Translation iModulons. (**A**) iModulonDB allows users to (1) search for iModulons; (2) select iModulons of interest and (3) analyse pairs of iModulons directly on the updated ‘Search’ page. iModulon-gene and gene-gene analyses are also available (not shown). The RpoS and Translation iModulon analysis in the *E. coli* PRECISE 1K dataset (17) is shown. iModulon-iModulon analysis will generate various graphs including (**B**) a plot of iModulon activities—known as a phase-plane—and condition-specific correlations between the activities of both iModulons; (**C**) a Venn diagram describing the genetic overlap between the two iModulons and a differential iModulon membership (DiMM) plot that displays the gene weights in each iModulon and (**D**) the protein-protein interaction network (all channels) retrieved from the STRING database for genes across both iModulons. We can identify that the two iModulons exhibit a moderate negative correlation (Panel B) but have no genes in common (Panel C). There exists a set of proteins that interact with gene-products from both iModulons (Panel D), suggesting potential avenues for further investigation. Samples from (Sastry *et al.* (27)) are highlighted in Panel B and show a decrease in global stress readiness for samples grown in rich media. Note: Sastry *et al.* studied antibiotic and media composition in the context of PRECISE-278 (1), a smaller *E. coli* dataset published along with the initial release of iModulonDB.

Further, this update describes iModulonDB’s transition from a static repository of curated iModulons to a dynamic knowledgebase that accommodates in-page analyses. Targeted analysis of any iModulon-iModulon, iModulon-gene, or gene-gene pair can be conducted through our updated ‘Search’ page (Figure 3). Dataset-wide analyses across all expression profiles of a species are also available on the new ‘Analysis’ page (Supplementary Figure S6). Here, all pairs of iModulons whose activities (or iModulon-regulator pairs whose activity and expression) are correlated above a user-defined threshold can be identified across the transcriptome. Similarly, iModulonDB can identify potential regulators for iModulons whose genes cannot be ascribed to any known regulon. Thus, these new analysis tools promote collaboration between iModulonDB users and the authors of the initial ICA decompositions to refine our characterization and understanding of iModulons as part of an iterative process. A list of all updates to iModulonDB can be found in Supplementary Table S1.

## Results

### Knowledgebase Content

At the time of its release, iModulonDB contained curated iModulon sets for three prokaryotic transcriptomic datasets: *E. coli* (1), *B. subtilis* (9), *S. aureus* (10). We have since downloaded, processed, computed, and published iModulon structures for all organisms with sufficient data on the Sequence Read Archive (16). To ensure the high-quality of these curated iModulon sets, ICA of the transcriptomic datasets was conducted using a consistent, publicly available pipeline (iModulonMiner (13) supported by a Python package for characterization, plotting, and other analyses (pymodulon (1)). These efforts have led to a dramatic expansion of iModulonDB, including a very high-quality single-protocol *E. coli* dataset with over 1000 samples (17) and datasets for twelve additional organisms: *A. baumannii* (2), *L. reuteri* (3), *M. tuberculosis* (11), *P. aeruginosa* (8,18), *P. putida* (19), *P. syringae* (4), *S. enterica* (20), *S. acidocaldarius* (5), *S. pyogenes* (6), *S. albidoflavus* (21), *S. elongatus* (7) and *V. natriegens* (22). The expansion of iModulonDB also includes additional publications of datasets for *B. subtilis* (13) and *S. aureus* (23). Currently, iModulonDB contains 1924 curated iModulons derived from 22 transcriptomic datasets spanning 9576 expression profiles and 247 publications (Table 1).

### Data curation facilitates understanding of underlying datasets

Knowledge of an experiment typically resides within its publication and associated metadata. To facilitate the transfer of this knowledge to our users, we have created a new ‘Projects’ page where the expression profiles (samples) in each ICA dataset are grouped by their respective study. Here, users can select a study from the drop-down menu to display: (1) the title, author, abstract, and DOI (when available); (2) the control and experimental variables and (3) a table that contains the sample ID and all relevant information that differs between the samples (Figure 1). In total, more than 240 studies in iModulonDB are accompanied by a publication whose abstract is displayed on the user-interface. In the example shown in Figure 1, the new ‘Projects’ page quickly directs the user to pertinent information about the ‘Acid’ study (24) in the *E. coli* PRECISE-1K dataset: *E. coli* MG1655 (*wildtype, ΔgadX, ΔgadW* or *ΔgadE*) grown aerobically in M9 medium at 37°C and pH of 5.5. By compiling and displaying key information about all experiments used to generate expression profiles in the ICA datasets, we establish iModulonDB as a knowledgebase and advance a key objective: to simplify access to and understanding of transcriptomic datasets.

As is the case in the ‘Acid’ study in *E. coli*, the data published in iModulonDB is often generated from experiments with genetically perturbed strains (gene are deleted, mutated, or expressed from a plasmid). In the initial release of iModulonDB, our dashboards displayed raw data, requiring the user to carefully interpret the associated graphs and correlations (e.g. between the expression of a gene regulator and the activity of the iModulon it regulates). To reduce the burden of interpretation on the user and to ensure the accuracy of correlations, we have further curated the metadata across all expression profiles in iModulonDB to explicitly describe the deletion, mutation, or vector expression of genes in each sample. When genetically perturbed samples directly affect the gene(s) or iModulon(s) plotted, these samples are highlighted but not used to calculate the correlations on the updated dashboards (Figure 2).

### Expanded toolkit for data visualization

Since the original publication of iModulons in *E. coli* (1), many graphical representations have been used to describe the iModulons and their underlying datasets. In this update, we describe a series of interactive graphical displays that help convey actionable information about iModulons. Each new graphic described here displays additional metadata ‘on hover’ and redirects to the appropriate page ‘on click’.

Independent component analysis (ICA) is a computational method used to separate a multivariate signal into additive, independent components. When applied to a transcriptomic dataset containing the expression profiles of all genes across multiple conditions, ICA decomposes the transcriptomic matrix into an *iModulon matrix* (independent signals, i.e. iModulons) and an *activity matrix* (intensity of each signal, i.e. activity of the iModulon in all conditions) (see Supplementary Figure S1). Limited by the number and diversity of the conditions included in the original transcriptomic matrix, ICA only maps a subset of genes to an iModulon (i.e. some genes are not sufficiently expressed in any condition). However, once iModulons are identified, the original transcriptomic matrix can be recreated using only genes belonging to the set of iModulons, and in doing so, the explained variance of the iModulon decomposition can be calculated. On each ‘Dataset’ page, a *TreeMap* is now used to rapidly convey this information (Supplementary Figure S3).

Unlike iModulons, *regulons* are sets of co-regulated genes identified through bottom-up biomolecular methods, which rely on the experimental measurement of transcription factor binding sites. While experimental validation of co-regulated genes is crucial for understanding an organism's transcriptional regulatory network (TRN), the high costs and condition-specific challenges associated with identifying binding sites have hindered efforts to experimentally validate the TRNs of lesser-studied organisms. Free from these constraints, iModulons offer an omics-driven, top-down approach that can either provide a global TRN for the first time (e.g. in *S. acidocaldarius* (5)) or highlight areas where our understanding of existing TRNs can be expanded. For example, in *E. coli*, our group previously identified the *MetJ iModulon*: a set of 17 co-expressed genes (*metA/BL/C/E/F/J/K/NIQ/R, hcxA, mmuMP, ybdL and yiiX*) in the PRECISE-278 dataset (1). Five of those genes (*hcxA, mmuMP, ybdL and yiiX*) were not included in the *MetJ regulon* (as described in RegulonDB (25)). ChIP-exo was then used to confirm the existence of MetJ binding sites for all five genes, thereby expanding the reported definition of the MetJ regulon. Similarly, the CysB iModulon also provided actionable insights that were experimentally validated (1).

To characterize differences between the experimentally determined TRN (i.e. from regulons) and the omics-driven TRN (i.e. from iModulons), *Regulon recall* (RR) and *iModulon recall* (MR) are used to quantify the overlap between the genes in a specific iModulon and those in its closely related regulon. In this update, we provide a *Recall Plot* on the ‘Dataset’ page to visualize the overlap between all iModulon-regulon pairs across an entire dataset (Supplementary Figure S3). Importantly, the ‘Regulon discovery’ quadrant of this plot identifies iModulons where experimental validation is likely to expand our understanding of the TRN for an organism. Furthermore, gene-level insights into iModulons and regulons can be derived from the gene expression correlation matrix displayed on each ‘iModulon’ page (see Supplementary Figure S4).

Transcriptional regulation is condition-specific, so we have implemented condition-specific highlighting for all data points in our dashboards. Previously, our dashboards displayed condition-specific behaviour for a gene or an iModulon, but did not include plots comparing iModulon activity and gene expression. This update introduces additional condition-specific correlation plots that accompany existing iModulon-regulator plots, as well as new plots showing an iModulon's activity and the expression of its constituent genes. These improvements allow users to see exactly which conditions contribute the most to correlations between iModulon activity and gene expression (e.g. Figure 2).

### Interoperability with external databases

Since the curation of iModulons is data-driven, researchers often seek a biological understanding of iModulon genes. A gene's operon structure and its protein-protein interaction (PPI) network may elucidate the biological mechanisms driving the gene's co-expression with other genes (and thus its inclusion in an iModulon). For example, our group previously found that an iModulon's gene membership can be explained by features inherent to the promoter sequences of its constituent genes (26). To promote the understanding of iModulons and their constituent genes, each ‘Gene’ page now contains direct links to a gene's operon(s) (via BioCyc (14)) and retrieves the protein-protein interaction (PPI) network (all channels) from the STRING database (15) (Supplementary Figure S5). The PPI networks of entire iModulons are also displayed on the ‘iModulon’ page and can be especially useful when an iModulon's genes are distributed across multiple operons. When selected, all PPI networks displayed on iModulonDB redirect to the STRING database where users can select specific channels (e.g. experimental evidence, co-expression, text-mining) to guide further iModulon characterization.

### Search-driven and species-wide analyses

To promote prolonged user engagement with iModulons and facilitate knowledge-mining of the underlying datasets, we developed a new set of features that enable in-page analyses (Figure 3A). The iModulon pairwise analysis feature allows users to select pairs of iModulons (via the ‘Dataset’ or the ‘Search’ page) and identify the conditions in which the activities of two iModulons are correlated. These pairwise activity plots—called *iModulon activity phase-planes—*reveal coordination or antagonism between iModulons and can offer insights into cellular phenotypes.

For example, the ‘fear-greed’ trade-off describes the balance between rapid cellular growth (i.e. ‘greed’) and stress response readiness (i.e. ‘fear’). Fear and greed phenotypes can be described by the activities of multiple iModulons. The *RpoS and GadEWX* iModulons contain genes involved in global stress response and acid-stress response, respectively, while the *Translation* and *ppGpp* iModulons contain ribosomal genes and genes that affect translation activity. In Figure 3B, samples from the ‘Antibiotics Media’ study (27) (*E. coli* MG1655 subject to different combinations of bacteriologic media and antibiotics) are displayed on the *RpoS vs Translation* iModulon phase-plane. We observe that rich media (RPMI & CA-MHB) leads to a strong decrease in *RpoS* iModulon activity. This reduction in stress-readiness frees up cellular resources, which can be allocated to promote growth (‘greed’), as evidenced by the increase in *Translation* iModulon activity. By contextualizing individual studies within a transcriptomic compendium, iModulon phase-planes can also elucidate the impact of specific mutations on global cellular responses or show evidence of an organism's adaptation to environmental conditions (see Dalldorf et al., 2024 (28)).

The new pairwise analysis workflow also yields additional graphs that supplement the phase-planes: a Venn diagram of the gene overlap (Figure 3C, inset), a scatter plot that highlights the differential gene membership (gene weights) between iModulons (Figure 3C), and the PPI network of genes from either iModulon (Figure 3D). Furthermore, analysis of any iModulon-gene or gene-gene pair can also be conducted on the updated ‘Search’ page.

In addition to user-specified analyses, the new ‘Analysis’ page of iModulonDB accommodates dataset-wide analyses across all expression profiles of a species (Supplementary Figure S6). Here, users select a desired R^2^ threshold to identify all pairs of iModulons or all iModulon-regulator pairs displaying the strongest correlations (activity-activity or activity-expression) in a specific dataset. In cases where iModulons cannot be mapped to a regulon (i.e. no overlapping genes), iModulonDB can now compare the activities of these iModulons to the expression of all mapped (to other iModulons) regulators and identify potential regulatory mechanisms (i.e. identify known regulators whose expression is correlated to ‘unregulated’ iModulons).

## Conclusion

Independent component analysis has proven to be a powerful tool for obtaining co-regulated, independently modulated gene sets from high-quality bacterial transcriptomic datasets. By contextualizing RNA-seq data from individual experiments within a broader transcriptomic compendium suitable for ICA, iModulons can uncover new layers of understanding from existing datasets. iModulonDB continues to be the most widely used and well-maintained platform for sharing and exploring iModulons. Although this update represents a 1370% growth in the number of expression profiles analysed and includes iModulon sets for 12 additional organisms, iModulonDB is no longer just a database. The collection and display of all publications and experimental variables, the interpretation of conditions and genetic perturbations, the biological context provided by external databases, and the user-prompted analysis tools now available establish iModulonDB as a dynamic knowledgebase that promotes understanding of bacterial transcriptomes and the condition-specific rules that govern regulatory relationships. iModulonDB is publicly available and continues to expand the content and functional capabilities within the knowledgebase. Driven by the needs of the research community, iModulonDB development has focused on disseminating curated iModulons (and the underlying transcriptomic datasets) in an easily interpretable manner, improving interoperability with external databases, and building analysis tools that enable users to interact directly with iModulons. To help shape future releases of iModulonDB, we encourage community members to submit feedback through the contact page.

## Supplementary Material

gkae1009_Supplemental_File

## Data Availability

iModulonDB is freely available online for academic and non-profit use at iModulonDB.org. Transcriptomic datasets hosted on iModulonDB can be downloaded directly or accessed through the original publications. The iModulonMiner package used to generate dashboards for iModulonDB is publicly available at https://github.com/SBRG/iModulonMiner and FigShare at the DOI (10.6084/m9.figshare.27229335). The link to access it is here (https://figshare.com/s/2d9cca12d9b8d111718c). We encourage community members to submit their own ICA decompositions and transcriptomic datasets using the contact information provided on our site.
