# Supplementary material for: iModulonDB 2.0: dynamic tools to facilitate knowledge-mining and user-enabled analyses of curated transcriptomic datasets
Source: Nucleic Acids Res. 2024 Nov 4;53(D1):D99–D106. doi: 10.1093/nar/gkae1009 (PMC11701608; doi:10.1093/nar/gkae1009)
Supplement: gkae1009_Supplemental_File [file gkae1009_supplemental_file.docx]

Supplementary Information for

iModulonDB 2.0: dynamic tools to facilitate knowledge-mining and user-enabled analyses of curated transcriptomic datasets

Edward A. Catoiu^1*^, Jayanth Krishnan^1^, Gaoyuan Li^1^, Xuwen A. Lou^1^, Kevin Rychel^1^, Yuan Yuan^1^, Heera Bajpe^1^, Arjun Patel^1^, Donghui Choe^1^, Jongoh Shin^1^, Joshua Burrows^1^, Patrick V. Phaneuf^2^, Daniel C. Zielinski^1^ & Bernhard O. Palsson^1,2^

^1^Department of Bioengineering, University of California, San Diego, La Jolla, CA 92101

^2^The Novo Nordisk Foundation (NNF) Center for Biosustainability, The Technical University of Denmark, Kongens Lyngby 2800, Denmark

* Correspondence to: ecatoiu@ucsd.edu

**This PDF file includes:**

**Table S1**

**Figures S1-S6**

All material can be found at [iModulonDB.org](http://imodulondb.org)

**Table S1: iModulonDB 2.0 contains updates across all pages.** All modifications and/or additions to the pages within iModulonDB are listed below. When available, figures from this study are referenced. Updates and new datasets are chronologically logged on our “What’s New” page ([imodulondb.org/update.html](https://imodulondb.org/update.html)). New pages are highlighted in green.

| **Page Name** | **Features & modifications in this update** |
| --- | --- |
| **Gene** | - PPI network (STRING) (Fig. S3) - Operon diagrams (BioCyc) (Fig. S3) - Gene expression vs iModulon activity graphs - Condition-specific highlighting & correlations |
| **iModulon** | - PPI network (STRING) (Fig. S3) - Gene expression correlation matrix heatmap (Fig. S2) - Gene expression correlation dendrogram - Condition-specific highlighting & correlations (vs Regulator) (Fig. 2) - Condition-specific highlighting & correlations (vs iM genes) (Fig. 2) |
| **Dataset** | - Explained variance TreeMap (Fig. S1) - Regulon recall plot (Fig. S1) - Pairwise iModulon analysis feature (Fig. 3) |
| **Projects**  (Fig. 1) | - Article metadata (title, authors, doi, and abstract) displayed - Control & experimental variables identified from sample data - Sample data table grouped by project - Dataset publication metadata is highlighted |
| **Analysis**  (Fig. S4) | - Pairwise iM-iM analysis (above R2 threshold) - Pairwise iM-regulator analysis (above R2 threshold) - Identification of potential regulators for iMs with no regulator (above R2 threshold) |
| **Search** | - Pairwise iM-iM analysis from search (Fig. 3) - Pairwise iM-gene analysis from search - Pairwise gene-gene analysis from search |
| **About** | - Modified “Using this Site” descriptions |
| **What’s New** | - New page to log updates and additional content |
| **Home** | - Database size dynamically calculated |
| **Backend** | - BioCyc SmartTables for all organisms to link gene IDs, names, external database links - BLASTed sequences to STRING to obtain correct identifier - Article metadata for 240+ DOIs - Additional datasets added (Table 1) - Dataset curation for genetically perturbed samples (used for updated analyses) |
| **General** | - Dynamic text descriptions implemented throughout each page |


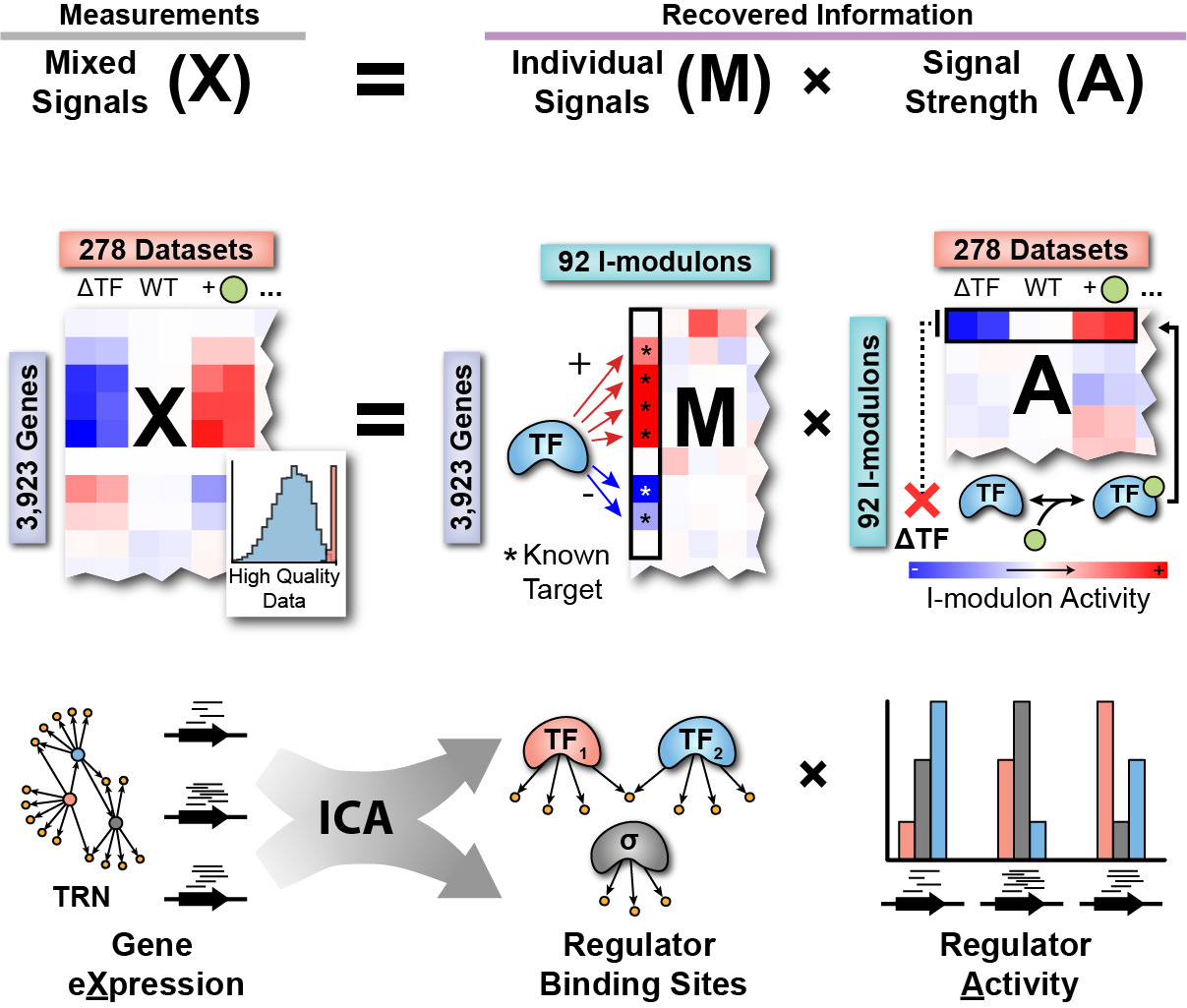


**Figure S1: Independent component analysis reveals independently modulated sets of genes within transcriptomic datasets.** (top) ICA is a computational method used to separate a multivariate signal into additive, independent components. (center) The transcriptomic matrix (in *E. coli* PRECISE-278^1^) is composed of the expression profiles of 3,923 genes across 278 experiments. ICA decomposes this matrix into the iModulon matrix (*M*) and activity matrix (*A*). Each *column* in *M* represents the weight of all genes in an iModulon. Each *row* in *A* represents the activity of an iModulon across all 278 experiments. (bottom) ICA identifies transcriptional regulators (and their activities across all conditions) from the compendium of individual expression profiles, revealing a modularized view of the transcription regulatory network. Published on the ‘About’ page with the initial release of iModulonDB, this figure is an adaptation of Figure 1 from Sastry et al. (2019).


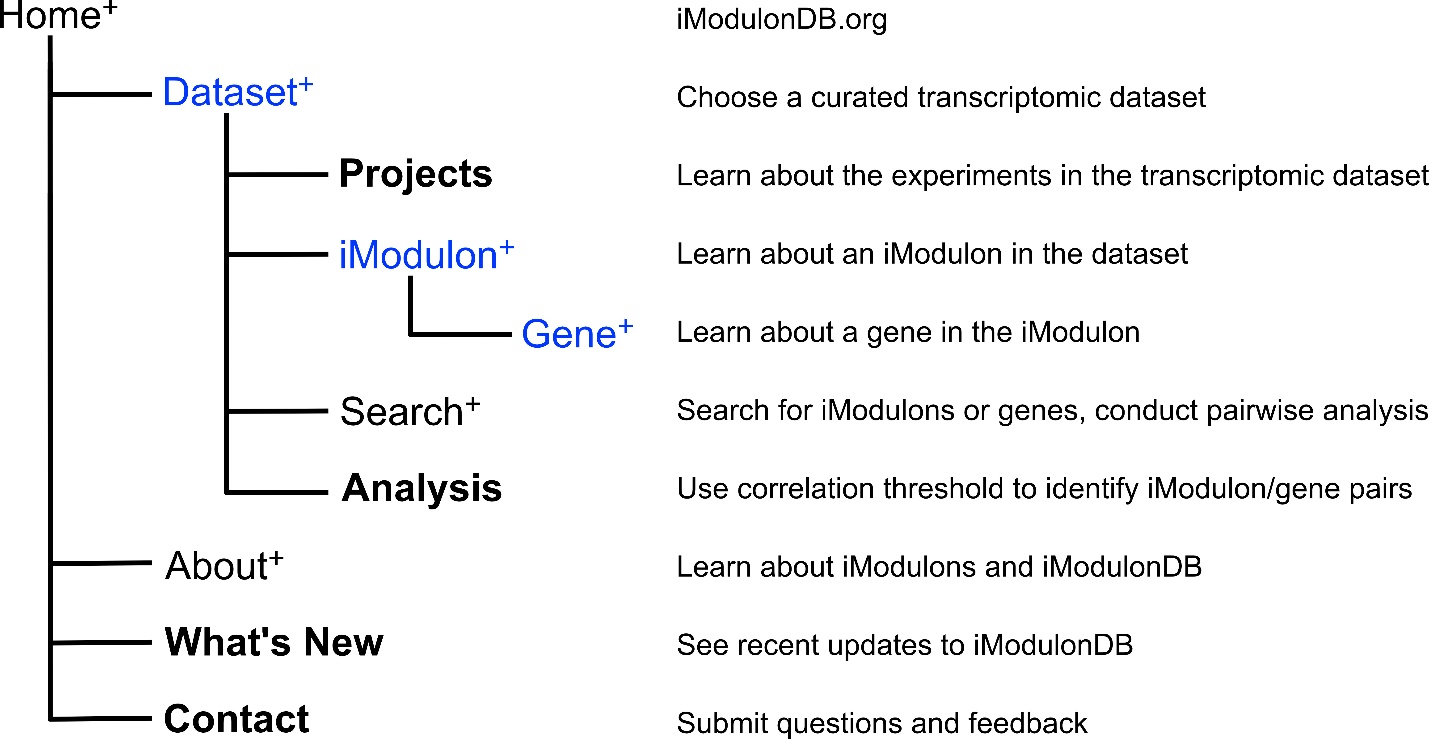


**Figure S2: Website architecture of iModulonDB.** New iModulonDB pages described in this update are shown in bold. New features on the user-interface are denoted by a “+”. Dashboard files generated by the iModulonMiner package^13^ are shown in blue. A complete list of updates can be found in Table S1. A more detailed explanation of graphs available in the initial release is available on our ‘About’ page and by Rychel et al. (2020).


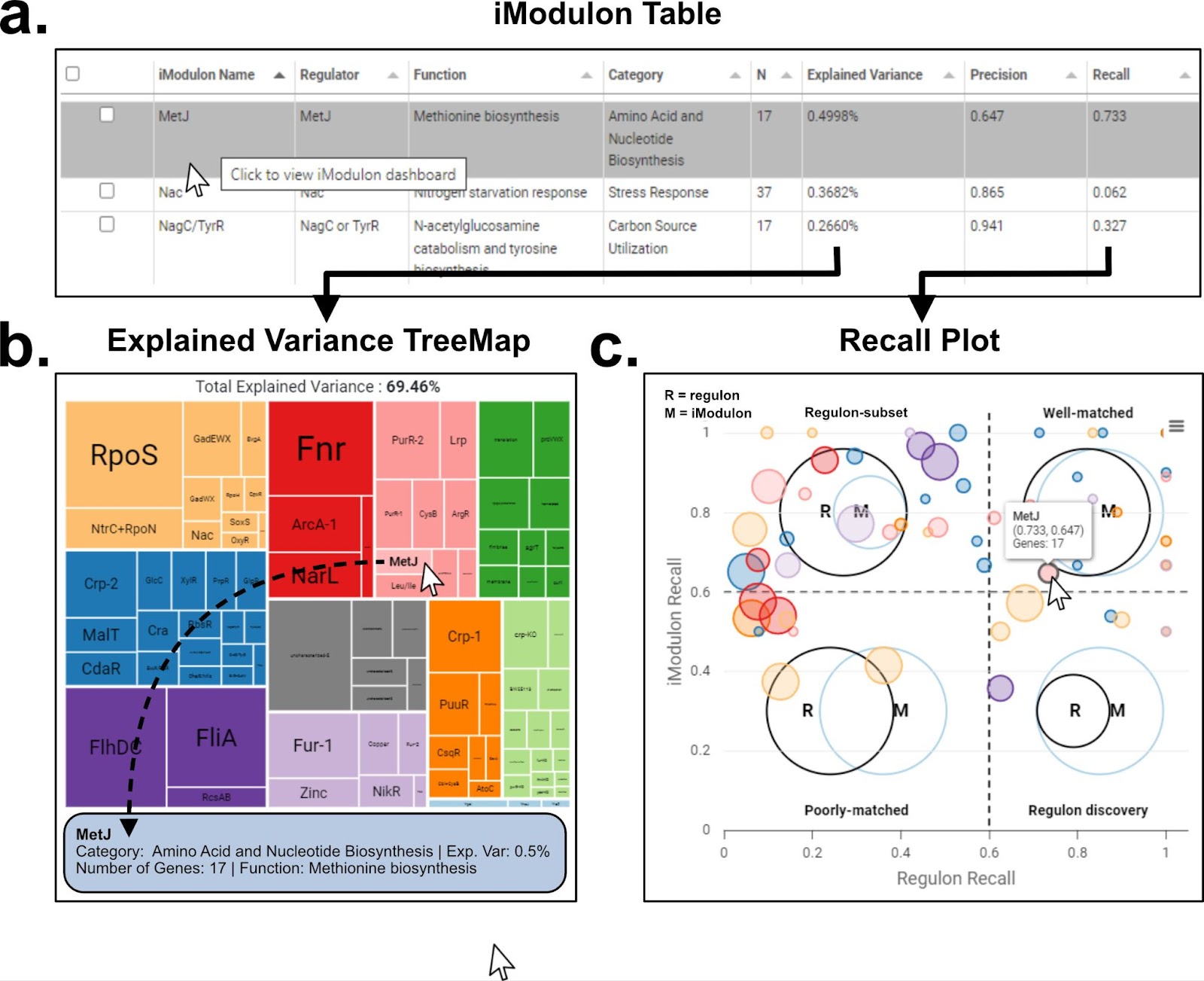


**Figure S3: TreeMaps and recall plots provide further characterization of each transcriptomic dataset of a species.** The original ‘Dataset’ page displayed **a)** the iModulon table which describes the name, function, size, explained variance and recall of each iModulon. From this table, **b)** the total explained variance is calculated and the per-iModulon variance is displayed as a TreeMap. When clicked, the boxes redirect to the corresponding ‘iModulon’ page. On hover, additional information about each iModulon is displayed below the TreeMap. **c)** The iModulon Recall (MR) and Regulon Recall (RR) are plotted, providing a visual assessment of possible TRN knowledge gaps for an organism. MR = shared genes / iModulon size. RR = shared genes / regulon size. The *E. coli* MetJ iModulon from the PRECISE-278 dataset^1^ (Sastry et al. 2019) is highlighted.


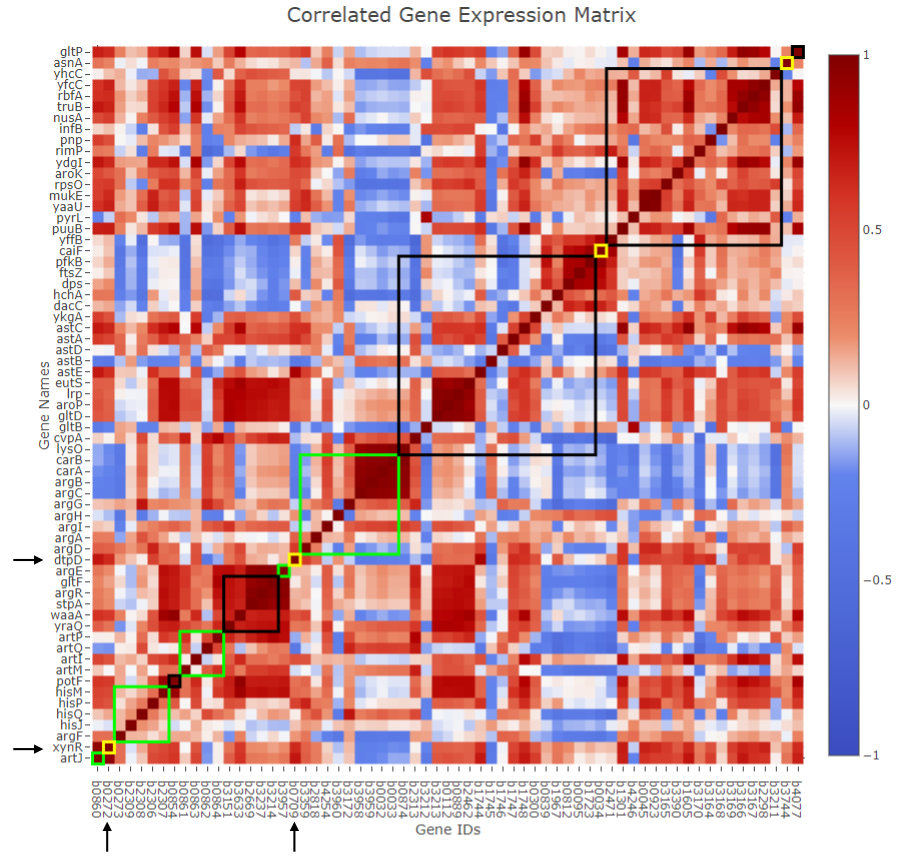


**Figure S4: The gene expression correlation matrix of the Arginine iModulon and regulon in PRECISE1K.** In this update, each ‘iModulon’ page displays a gene expression correlation heatmap to analyze relationships between the iModulon and its associated regulons. The heatmap is based on Pearson correlations of genes from both iModulons (yellow) and linked regulons (black), reveals the co-expression patterns, and illustrates the portions of the regulon that are effectively captured by the iModulon (green). Notably, genes exclusive to the iModulon often show co-expression patterns with the regulon components recovered by the iModulon. For instance, *xynR* and *dtpD* (arrows), absent from the Arginine regulon but identified by the iModulon, show high correlation with the regulon components. This visualization validates iModulon predictions and suggests potential expansion of the regulon. Additionally, it aids in refining iModulon composition by identifying genes that may not correlate strongly with the main group, improving the accuracy of iModulon definitions.


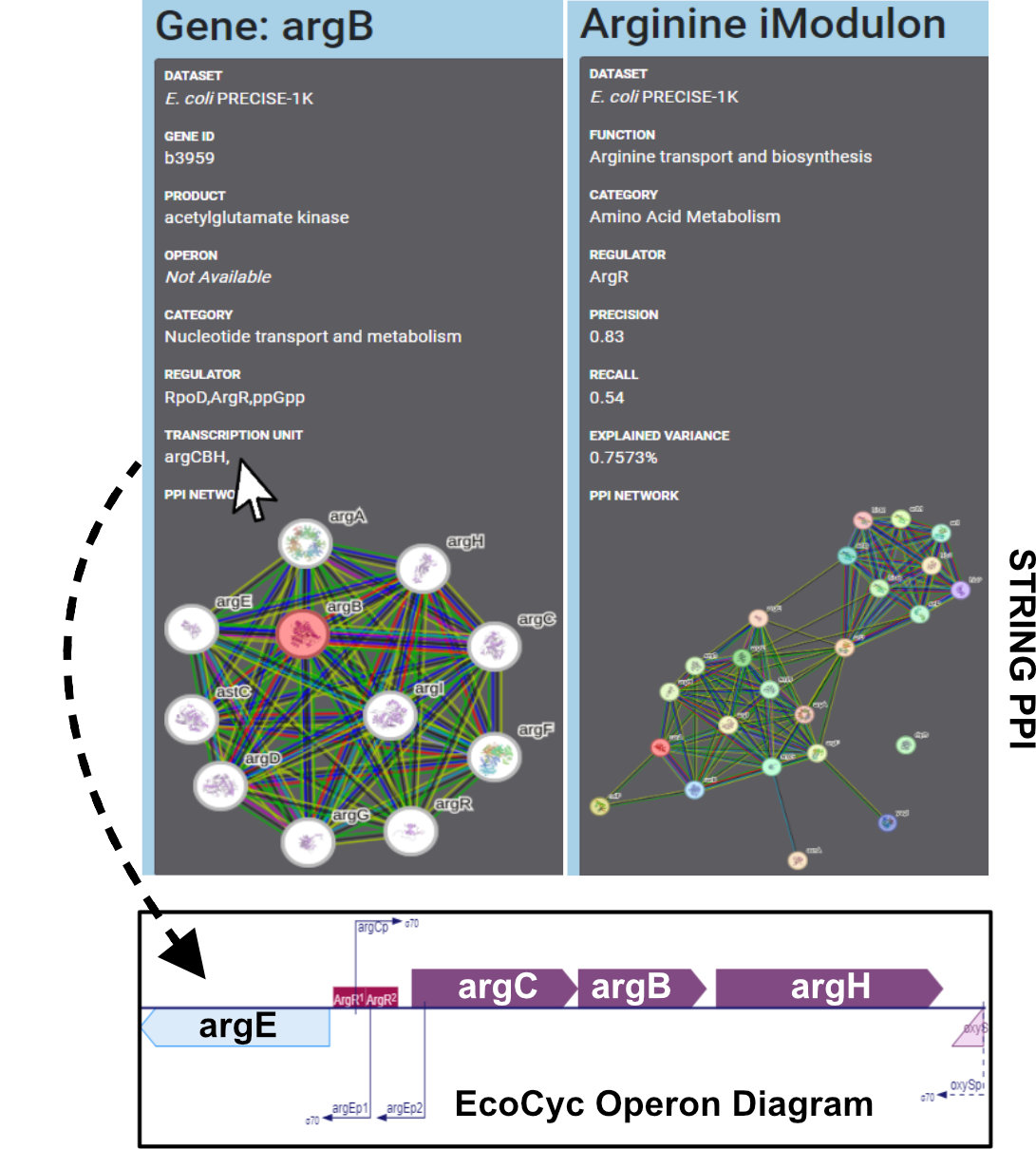


**Figure S5: Interoperability with BioCyc and STRING databases.** (left) The header of each ‘Gene’ page contains links to the operon diagram of a gene (BioCyc^14^) and displays the full PPI network (STRING^15^). The header of each ‘iModulon’ page displays the PPI network (STRING) for a query of all genes in the iModulon. Both PPI graphs redirect to the STRING database when clicked.


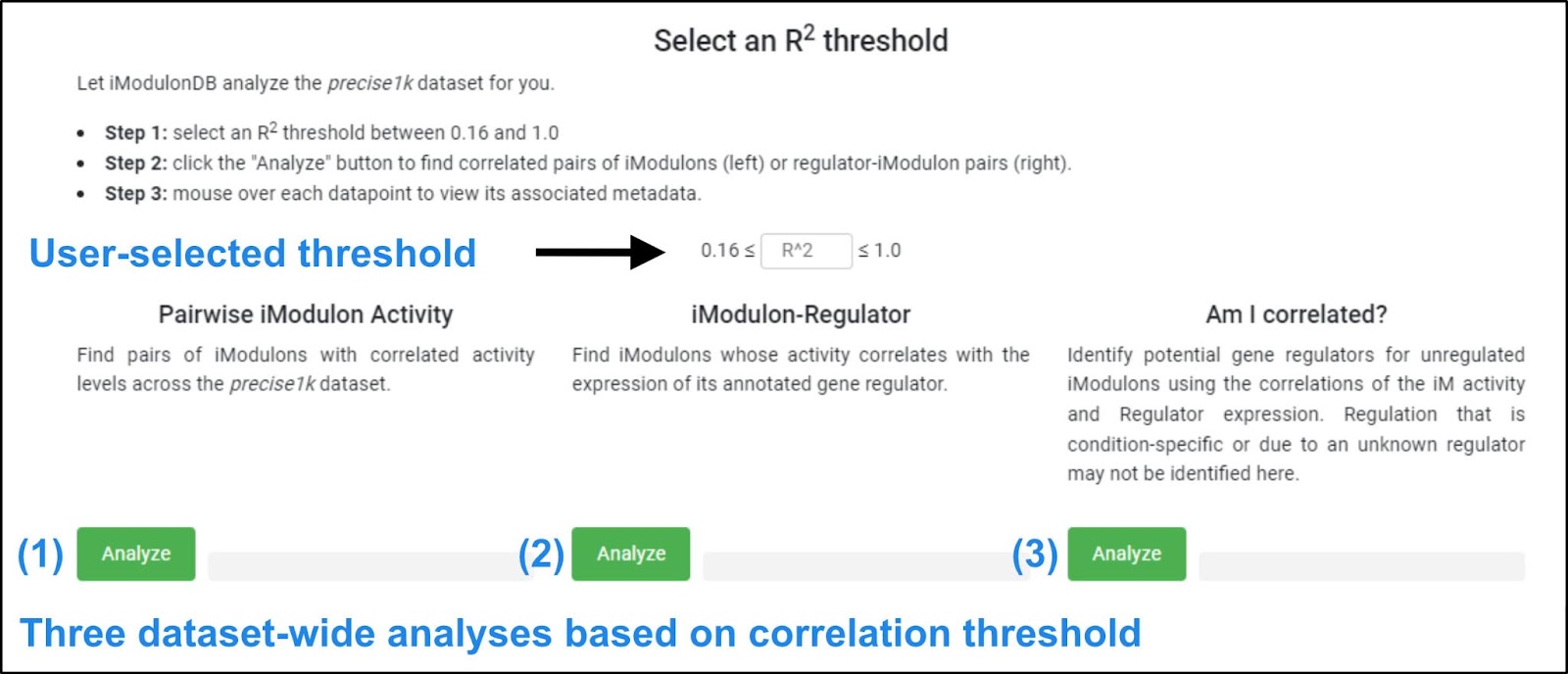


**Figure S6: The new ‘Analysis’ page offers three species-wide analysis tools.** Users are prompted to select a correlation threshold. Across all expression profiles of a species (within a transcriptomic dataset): 1) pairs of iModulons whose activities are correlated above the threshold will be identified across a dataset; 2) iModulons whose activities are correlated to the expression of its regulator will be identified across a dataset; and 3) known regulators whose expression correlates to iModulons without a known regulator can be identified.  Each analysis provides at least activity-activity or activity-expression plots and condition-specific correlation plots.
